# Profile of patients diagnosed with acute venous thromboembolism in routine practice according to age and renal function: RE-COVERY DVT/PE study

**DOI:** 10.1007/s11239-020-02239-9

**Published:** 2020-08-26

**Authors:** Walter Ageno, Ivan B. Casella, Kok Han Chee, Sebastian Schellong, Sam Schulman, Daniel E. Singer, Marc Desch, Wenbo Tang, Isabelle Voccia, Kristina Zint, Samuel Z. Goldhaber

**Affiliations:** 1 Department of Medicine and Surgery, University of Insubria, Via Guicciardini 9, 21100 Varese, Italy; 2 University of São Paulo, São Paulo, Brazil; 3 University of Malaya, Kuala Lumpur, Malaysia; 4 Städtisches Klinikum Dresden, Dresden, Germany; 5 Thrombosis and Atherosclerosis Research Institute and McMaster University, Hamilton, ON Canada; 6 Department of Obstetrics and Gynecology, The I.M. Sechenov First Moscow State Medical University, Moscow, Russia; 7 Massachusetts General Hospital and Harvard Medical School, Boston, MA USA; 8 Boehringer Ingelheim International GmbH, Ingelheim am Rhein, Germany; 9 Boehringer Ingelheim Pharmaceuticals Inc., Ridgefield, CT USA; 10 Boehringer Ingelheim Canada, Burlington, ON Canada; 11 Brigham and Women’s Hospital and Harvard Medical School, Boston, MA USA

**Keywords:** Anticoagulation, Elderly, Nonvitamin K antagonist oral anticoagulant, Renal function, Vitamin K antagonist

## Abstract

**Abstract:**

In randomized clinical trials (RCTs) of nonvitamin K antagonist oral anticoagulants (NOACs) for acute venous thromboembolism (VTE), ~ 12–13% of patients were elderly and ~ 26% had mild-to-moderate renal impairment. Observational studies are not restricted by the selection and treatment criteria of RCTs. In this ancillary analysis of the RE-COVERY DVT/PE global observational study, we aimed to describe patient characteristics, comorbidities, and anticoagulant therapy for subgroups of age (< or ≥ 75 years) and renal impairment (creatinine clearance [CrCl; estimated with Cockcroft–Gault formula] < 30 [severe], 30 to < 50 [moderate], 50 to < 80 [mild], ≥ 80 [normal] mL/min). Of 6095 eligible patients, 25.3% were aged ≥ 75 years; 38.2% (1605/4203 with CrCl values) had mild-to-moderate renal impairment. Comorbidities were more common in older patients (73.9% aged ≥ 75 vs. 58.1% < 75 years) and in those with mild or moderate versus no renal impairment (75.9%, 80.9%, and 59.3%, respectively). At hospital discharge or 14 days after diagnosis (whichever was later), most patients (53.7% and 55.1%, respectively) in both age groups received NOACs; 20.8% and 23.4%, respectively, received vitamin K antagonists, 19.0% and 21.8% parenteral therapy, 2.3% and 3.8% other anticoagulant treatments. Use of NOACs decreased with worsening renal impairment (none 58.5%, moderate 49.6%, severe 25.7%) and, in younger versus older patients with moderate renal impairment (33.1% vs. 56.1%). In routine practice, there are more elderly and renally impaired patients with VTE than represented in RCTs. Decreasing renal function, but not older age, was associated with less NOAC use. Clinical Trial Registration: http://www.clinicaltrials.gov. Unique identifier: NCT02596230.

**Graphic abstract:**

Decreasing renal function, particularly in the subgroup with CrCl < 30 mL/min, but not older age, was associated with less use of nonvitamin K antagonist oral anticoagulants (NOACs). Nevertheless, more than half of the older patients with moderate renal impairment received a NOAC as their oral anticoagulant.
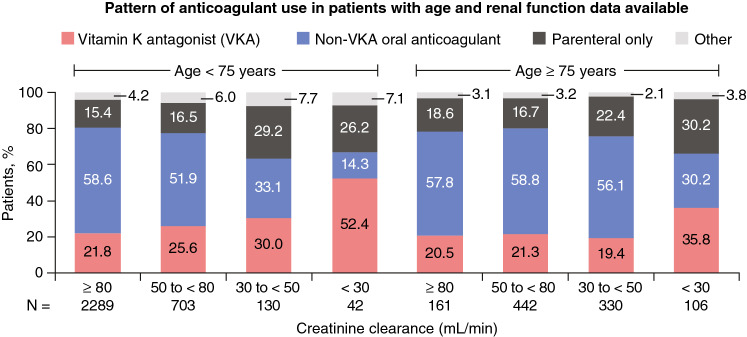

## Highlights

- RE-COVERY DVT/PE was a study of acute venous thromboembolism treatment in routine clinical practice.
- Compared with randomized clinical trials, more patients were elderly and renally impaired.
- Over half of the patients received nonvitamin K antagonist oral anticoagulants (NOACs), suggesting an increased confidence with these drugs in elderly populations.
- Decreasing renal function, but not older age, was associated with less NOAC use.
- The observed approach is supported by the results of randomized clinical trials that showed a better safety profile of NOACs compared with VKAs, particularly in elderly patients.

## Introduction

The risk of venous thromboembolic events (VTEs), comprising deep vein thrombosis (DVT) and/or pulmonary embolism (PE), increases with age [1] and chronic kidney disease [2]. Sparse data are available to help guide clinicians in choosing the optimal anticoagulation regimens for elderly or renally impaired patients, who are often excluded from randomized clinical trials (RCTs).

Current guidelines suggest the use of nonvitamin K antagonist oral anticoagulants (NOACs) over vitamin K antagonists (VKAs) for the treatment of acute VTE [3], based on the similar efficacy and improved safety of NOACs versus VKAs in the RCT patient populations [4–7]. However, the guidelines [3] do not provide specific recommendations for treating elderly and/or renally impaired patients, but the product labels for NOACs provide specific recommendations for dose adjustment in these patients [8–10].

The RE-COVERY DVT/PE study has two objectives. In the first phase, it aims to characterize patients with VTE, the location of VTE events, and treatment patterns at initial presentation [11]. In the second phase, it evaluates outcomes in patients treated with dabigatran or VKA in routine clinical practice [12]. We previously described baseline characteristics of the overall cohort of patients in the first phase, regional variations in treatment choices, and the influence of baseline clinical features such as cancer on the choice of anticoagulants [11]. Overall, 77% of patients received oral anticoagulants (54% NOACs and 23% VKAs), and 20% received parenteral anticoagulation only. NOACs comprised about 60% of anticoagulant treatment in Europe and Asia but less than a third in Latin America and the Middle East. The proportion of patients treated with NOACs was lower among those with cancer, chronic renal disease, heart failure, or stroke than in those without these comorbidities. Irrespective of the index event (DVT, PE, DVT, and PE), NOACs were the most common choice of anticoagulants. However, analysis of standardized differences suggested there was lower use of NOACs in patients with co-existing DVT and PE (49.5%) compared with DVT alone (54.9%). The pattern of NOAC use did not vary notably according to location of DVT (distal, proximal, upper limb, or other).

In the current report using demographic data from the first phase of the study, we evaluate the profile of patients treated for acute VTE in routine clinical practice according to their age and renal function and to compare it, where feasible, with the profiles of patients in randomized clinical trials of dabigatran. Furthermore, we also aimed to investigate the anticoagulant treatment strategies used in the different age and renal function subgroups.

## Methods

### Study design

The rationale and design of the large, multicenter, international RE-COVERY DVT/PE observational study have been described previously [12]. In the first phase, patients with acute VTE were characterized according to baseline features and initial treatment. Patients could be entered into the study up to 6 months following the acute event. Investigators were encouraged to include consecutive patients with acute VTE, irrespective of initial treatment. In the second phase, safety and effectiveness outcomes over a follow-up period of 1 year were compared for dabigatran and VKA.

The study was carried out in compliance with the protocol and the principles laid down in the Declaration of Helsinki. In addition, the applicable sections of the guidelines for Good Clinical Practice, Good Epidemiological Practice, and Good Pharmacoepidemiology Practices, and local regulations were followed. Patients (or their legal representative) provided written informed consent before study entrance, in accordance with local regulations. No study procedures or data recording were performed unless a patient had consented to participate in the study or a waiver had been obtained in accordance with local regulations.

### Eligibility criteria

Eligible patients included those aged ≥ 18 years and diagnosed with acute proximal or distal DVT and/or PE. If anticoagulation was required for any condition other than VTE, or if patients were participating in another clinical trial for VTE, they were excluded.

### Data collection

Following a diagnosis of VTE, patient characteristics and anticoagulant treatment administered at baseline were recorded. As treatment with some oral anticoagulants may be preceded by parenteral anticoagulation with heparin or fondaparinux, anticoagulant therapy was recorded again at hospital discharge or at 14 days after the diagnosis, whichever was later. Sites recorded all clinical data and site/investigator characteristics via a secure, web-based, electronic data capture system. Potential selection bias was minimized by using consecutive, unselected enrollment (regardless of a patient’s treatment or management).

### Statistical analysis

Approximately 6000 patients were planned to be enrolled in phase 1 of the study. The sample size was not based on formal sample size calculations, as no a priori hypothesis testing was involved. Based on a range of prevalence rates of events or patient attributes and the width of the associated 95% confidence intervals, a sample size of 6000 was considered reasonable. Data obtained at baseline (patient characteristics, hospitalization details, and anticoagulant therapy) were tabulated according to age (< 75 and ≥ 75 years) and renal function (creatinine clearance [CrCl] estimated using the Cockcroft–Gault formula: < 30 mL/min [severe impairment], 30 to < 50 mL/min [moderate impairment], 50 to < 80 mL/min [mild impairment], and ≥ 80 mL/min [normal]). The assignment of treatment choice was based on data from hospital discharge or 14 days after diagnosis (whichever was later). As such, patients who received parenteral anticoagulation prior to, or overlapping with, oral anticoagulation were considered to have been treated with the relevant oral anticoagulant.

## Results

From January 13, 2016 to May 4, 2017, 6194 patients were consecutively enrolled from 34 countries, of whom 6095 patients were eligible. Ninety-nine patients were excluded: 46 patients had no documented VTE treatment; 29 had issues with the informed consent form; and 24 did not satisfy inclusion/exclusion criteria (lack of written informed consent, n = 3; lack of diagnosis of acute VTE/PE, n = 16; age < 18 years, n = 1; anticoagulation indicated for conditions other than VTE, n = 4).

### Baseline characteristics and anticoagulation treatment of eligible patients according to age or renal function

Baseline characteristics are summarized in Table 1 for all 6095 eligible patients according to age, and for the 4203 patients who had CrCl data according to renal function. One-quarter of patients at baseline were aged ≥ 75 years, of whom nearly two-thirds were female. Of the 4507 patients diagnosed with DVT in the lower limb (with or without PE), 2819 (62.5%) had a proximal location (popliteal vein and above) and 2468 (54.8%) had a distal location (more than one location was possible). In patients aged < 75 years, 46.1% were female. A greater proportion of older patients (compared with those aged < 75 years) had renal impairment (mild 28.7% vs. 15.4%; moderate 21.4% vs. 2.9%; or severe 6.9% vs. 0.9%), PE as the index VTE event (32.4% vs. 23.9%), and comorbidities (73.9% vs. 58.1%), the most common being hypertension, diabetes mellitus, cancer, and a history of VTE.

**Table 1:** Baseline demographic characteristics according to age or renal function

	Age, years		CrCl,^b^ mL/min				All patients N = 6095
	< 75 n = 4553	≥ 75 n = 1542	≥ 80 n = 2450	50 to < 80 n = 1145	30 to < 50 n = 460	< 30 n = 148	
Male,^a^ n (%)	2453 (53.9)	609 (39.5)	1367 (55.8)	542 (47.3)	146 (31.7)	45 (30.4)	3062 (50.2)
Age, years, mean ± SD	54.7 ± 14.0	81.6 ± 5.1	53.5 ± 14.9	70.3 ± 10.9	78.1 ± 10.0	78.8 ± 14.1	61.5 (17.0)
Age group, n (%)							
< 75	4553 (100.0)	0	2289 (93.4)	703 (61.4)	130 (28.3)	42 (28.4)	4553 (74.7)
≥ 75	0	1542 (100.0)	161 (6.6)	442 (38.6)	330 (71.7)	106 (71.6)	1542 (25.3)
CrCl,^b^ mL/min, mean ± SD	106.9 ± 44.2	57.1 ± 23.9	122.7 ± 38.7	65.4 ± 8.6	41.2 ± 5.6	21.9 ± 6.6	94.6 (45.7)
CrCl class, mL/min, n (%)							
< 30	42 (0.9)	106 (6.9)	0	0	0	148 (100.0)	148 (2.4)
30 to < 50	130 (2.9)	330 (21.4)	0	0	460 (100.0)	0	460 (7.5)
50 to < 80	703 (15.4)	442 (28.7)	0	1145 (100.0)	0	0	1145 (18.8)
≥ 80	2289 (50.3)	161 (10.4)	2450 (100.0)	0	0	0	2450 (40.2)
Missing	1389 (30.5)	503 (32.6)	0	0	0	0	1892 (31.0)
BMI,^c,d^ kg/m^2^, mean ± SD	28.5 ± 6.4	26.8 ± 4.9	29.8 ± 6.7	26.6 ± 4.8	25.5 ± 4.7	24.9 ± 5.7	28.1 (± 6.1)
Index VTE event, n (%)							
DVT	2788 (61.2)	856 (55.5)	1383 (56.4)	611 (53.4)	223 (48.5)	89 (60.1)	3644 (59.8)
PE	1088 (23.9)	500 (32.4)	614 (25.1)	330 (28.8)	148 (32.2)	44 (29.7)	1588 (26.1)
DVT and PE	677 (14.9)	186 (12.1)	453 (18.5)	204 (17.8)	89 (19.3)	15 (10.1)	863 (14.2)
Clinical features,^e^ n (%)							
None	1909 (41.9)	403 (26.1)	996 (40.7)	276 (24.1)	88 (19.1)	26 (17.6)	2312 (37.9)
Any	2644 (58.1)	1139 (73.9)	1454 (59.3)	869 (75.9)	372 (80.9)	122 (82.4)	3783 (62.1)
Hypertension	1325 (29.1)	788 (51.1)	726 (29.6)	580 (50.7)	262 (57.0)	83 (56.1)	2113 (34.7)
Diabetes mellitus	482 (10.6)	214 (13.9)	237 (9.7)	175 (15.3)	68 (14.8)	20 (13.5)	696 (11.4)
Cancer^f^	474 (10.4)	199 (12.9)	219 (8.9)	169 (14.8)	65 (14.1)	15 (10.1)	673 (11.0)
History of VTE	507 (11.1)	163 (10.6)	307 (12.5)	121 (10.6)	38 (8.3)	22 (14.9)	670 (11.0)
Trauma or surgery	325 (7.1)	123 (8.0)	211 (8.6)	88 (7.7)	38 (8.3)	10 (6.8)	448 (7.4)
Coronary artery disease	178 (3.9)	140 (9.1)	104 (4.2)	94 (8.2)	46 (10.0)	18 (12.2)	318 (5.2)
Chronic renal disease	121 (2.7)	102 (6.6)	27 (1.1)	36 (3.1)	53 (11.5)	53 (35.8)	223 (3.7)
Heart failure	98 (2.2)	86 (5.6)	43 (1.8)	62 (5.4)	35 (7.6)	12 (8.1)	184 (3.0)
Varicose veins	148 (3.3)	40 (2.6)	90 (3.7)	42 (3.7)	10 (2.2)	0	188 (3.1)
Stroke	95 (2.1)	72 (4.7)	52 (2.1)	45 (3.9)	19 (4.1)	15 (10.1)	167 (2.7)
Immobilization	115 (2.5)	42 (2.7)	81 (3.3)	20 (1.7)	9 (2.0)	6 (4.1)	157 (2.6)
Myocardial infarction	80 (1.8)	67 (4.3)	46 (1.9)	43 (3.8)	16 (3.5)	9 (6.1)	147 (2.4)
Atrial fibrillation	60 (1.3)	76 (4.9)	33 (1.3)	41 (3.6)	24 (5.2)	9 (6.1)	136 (2.2)

*BMI* body mass index, *CrCl* creatinine clearance, *DVT* deep vein thrombosis, *PE* pulmonary embolism, *SD* standard deviation, *VTE* venous thromboembolic event

^a^Information on sex missing for one patient aged < 75 years

^b^CrCl data missing for 1892 patients: 1389 patients aged < 75 years and 503 patients aged ≥ 75 years. CrCl estimated using the Cockcroft–Gault formula: < 30 mL/min represents severe impairment, 30 to < 50 mL/min moderate impairment, 50 to < 80 mL/min mild impairment and ≥ 80 mL/min normal

^c^BMI data missing for 934 patients aged < 75 years and 417 patients aged ≥ 75 years

^d^BMI data missing for 17 patients with CrCl < 30 mL/min, 44 patients with CrCl 30 to < 50 mL/min, 102 patients with CrCl 50 to < 80 mL/min and 210 patients with CrCl ≥ 80 mL/min

^e^Comorbidities and/or medical history present in ≥ 2% of patients overall are shown individually

^f^Excluding nonmelanoma skin cancer

Of the 4203 patients with known CrCl values, 38.2% had mild-to-moderate renal impairment (CrCl 30 to < 80 mL/min) and 3.5% had CrCl < 30 mL/min (Table 1). The percentage of male patients declined with decreasing renal function (55.8% for those with CrCl ≥ 80 mL/min to 30.4% for those with CrCl < 30 mL/min). With declining renal function from normal to moderate impairment, the proportion of patients with DVT alone appeared to decrease slightly (from 56.4% to 48.5%), and PE alone increased slightly (from 25.1% to 32.2%). The severe renal impairment group had fewer patients with both DVT and PE (10.1% compared with 17.8% to 19.3% in the other subgroups) and more with DVT alone (60.1% vs. 48.5% to 53.4%). More patients with mild, moderate, or severe renal impairment had comorbidities (75.9%, 80.9%, and 82.4%, respectively) compared with those with CrCl ≥ 80 mL/min (59.3%).

**Fig. 1:**
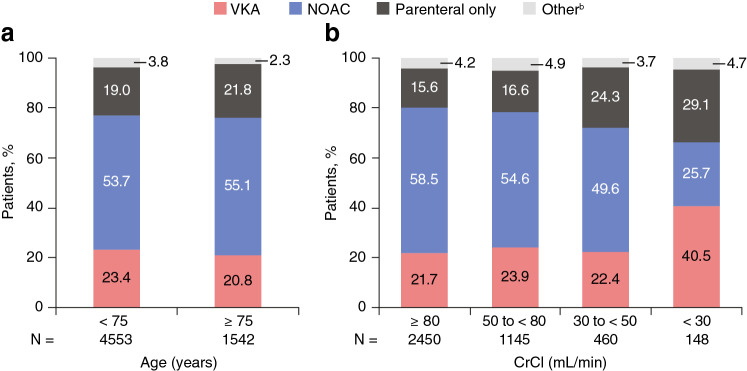
Pattern of anticoagulant use at hospital discharge or 14 days after diagnosis (whichever was later) according to **a** age or **b** renal function. ^a^*CrCl* creatinine clearance, *NOAC* non-VKA oral anticoagulant, *VKA* vitamin K antagonist. ^a^CrCl data missing for 1892 patients. CrCl estimated using the Cockcroft–Gault formula: < 30 mL/min represents severe impairment, 30 to < 50 mL/min moderate impairment, 50 to < 80 mL/min mild impairment, and ≥ 80 mL/min normal. ^b^“Other” includes catheter-directed or systemic thrombolytic therapy

At the time of hospital discharge or 14 days after diagnosis (whichever was later), most patients were treated with NOACs (54.0%). VKAs were prescribed to about 1 in 5 patients, and a similar proportion received parenteral anticoagulation only. Age, above or below 75 years, had minimal impact on the anticoagulation prescription pattern (Fig. 1a). Use of NOACs decreased with worsening renal function, particularly in the subgroup with CrCl < 30 mL/min. The corollary was greater use of parenteral therapy alone (24.3% to 29.1% in the moderate and severe impairment groups vs. 15.6% to 16.6% in the normal and mild impairment groups) and greater use of VKAs (40.5% in the severe impairment group vs. 21.7% to 23.9% in the normal, moderate, and mild impairment groups) (Fig. 1b).

### Baseline characteristics and anticoagulation treatment of patients with both age and renal function data available

Among the 4203 patients who had age and renal function data available, characteristics of the combined age/renal function subgroups (Table 2) generally reflected the patterns reported above for the separate age and renal function comparisons.

**Table 2:** Baseline demographic characteristics in 4203 patients with age and renal function data available

CrCl (mL/min)	Patients aged < 75 years with renal function data (N = 3164)				Patients aged ≥ 75 years with renal function data (N = 1039)			
	≥ 80 n = 2289	50 to < 80 n = 703	30 to < 50 n = 130	< 30 n = 42	≥ 80 n = 161	50 to < 80 n = 442	30 to < 50 n = 330	< 30 n = 106
Male,^a^ n (%)	1288 (56.3)	344 (48.9)	51 (39.2)	20 (47.6)	79 (49.1)	198 (44.8)	95 (28.8)	25 (23.6)
Age, years, mean ± SD	51.7 ± 13.8	64.1 ± 8.9	65.6 ± 8.5	60.9 ± 13.0	78.7 ± 3.1	80.3 ± 4.4	83.1 ± 4.9	86.0 ± 5.8
CrCl,^b^ mL/min, mean ± SD	124.4 ± 39.1	66.9 ± 8.5	42.6 ± 5.7	18.8 ± 7.2	97.1 ± 19.9	63.1 ± 8.2	40.6 ± 5.5	23.2 ± 5.9
BMI,^c,d^ kg/m^2^, mean ± SD	29.8 ± 6.8	26.2 ± 4.8	25.0 ± 5.0	26.7 ± 7.6	29.7 ± 5.5	27.3 ± 4.6	25.7 ± 4.6	24.1 ± 4.4
Index VTE event, n (%)								
DVT	1295 (56.6)	387 (55.0)	65 (50.0)	26 (61.9)	88 (54.7)	224 (50.7)	158 (47.9)	63 (59.4)
PE	566 (24.7)	182 (25.9)	32 (24.6)	12 (28.6)	48 (29.8)	148 (33.5)	116 (35.2)	32 (30.2)
DVT and PE	428 (18.7)	134 (19.1)	33 (25.4)	4 (9.5)	25 (15.5)	70 (15.8)	56 (17.0)	11 (10.4)
Clinical features,^e^ n (%)								
None	951 (41.5)	183 (26.0)	25 (19.2)	4 (9.5)	45 (28.0)	93 (21.0)	63 (19.1)	22 (20.8)
Any	1338 (58.5)	520 (74.0)	105 (80.8)	38 (90.5)	116 (72.0)	349 (79.0)	267 (80.9)	84 (79.2)
Hypertension	637 (27.8)	339 (48.2)	66 (50.8)	25 (59.5)	89 (55.3)	241 (54.5)	196 (59.4)	58 (54.7)
Diabetes mellitus	217 (9.5)	109 (15.5)	24 (18.5)	10 (23.8)	20 (12.4)	66 (14.9)	44 (13.3)	10 (9.4)
Cancer^f^	197 (8.6)	105 (14.9)	27 (20.8)	4 (9.5)	22 (13.7)	64 (14.5)	38 (11.5)	11 (10.4)
History of VTE	291 (12.7)	64 (9.1)	8 (6.2)	5 (11.9)	16 (9.9)	57 (12.9)	30 (9.1)	17 (16.0)
Trauma or surgery	194 (8.5)	50 (7.1)	9 (6.9)	4 (9.5)	17 (10.6)	38 (8.6)	29 (8.8)	6 (5.7)
Coronary artery disease	86 (3.8)	53 (7.5)	7 (5.4)	5 (11.9)	18 (11.2)	41 (9.3)	39 (11.8)	13 (12.3)
Chronic renal disease	27 (1.2)	23 (3.3)	22 (16.9)	24 (57.1)	0	13 (2.9)	31 (9.4)	29 (27.4)
Heart failure	38 (1.7)	29 (4.1)	7 (5.4)	6 (14.3)	5 (3.1)	33 (7.5)	28 (8.5)	6 (5.7)
Varicose veins	81 (3.5)	25 (3.6)	1 (0.8)	0	9 (5.6)	17 (3.8)	9 (2.7)	0
Stroke	46 (2.0)	24 (3.4)	6 (4.6)	4 (9.5)	6 (3.7)	21 (4.8)	13 (3.9)	11 (10.4)
Immobilization	74 (3.2)	8 (1.1)	2 (1.5)	2 (4.8)	7 (4.3)	12 (2.7)	7 (2.1)	4 (3.8)
Myocardial infarction	32 (1.4)	24 (3.4)	4 (3.1)	4 (9.5)	14 (8.7)	19 (4.3)	12 (3.6)	5 (4.7)
Atrial fibrillation	26 (1.1)	18 (2.6)	3 (2.3)	1 (2.4)	7 (4.3)	23 (5.2)	21 (6.4)	8 (7.5)

*BMI* body mass index, *CrCl* creatinine clearance, *DVT* deep vein thrombosis, *PE* pulmonary embolism, *SD* standard deviation, *VTE* venous thromboembolic event

^a^Information on sex missing for one patient aged < 75 years

^b^CrCl data missing for 1892 patients: 1389 patients aged < 75 years and 503 patients aged ≥ 75 years. CrCl estimated using the Cockcroft–Gault formula: < 30 mL/min represents severe impairment, 30 to < 50 mL/min moderate impairment, 50 to < 80 mL/min mild impairment and ≥ 80 mL/min normal

^c^BMI data missing for 934 patients aged < 75 years and 417 patients aged ≥ 75 years

^d^BMI data missing for 17 patients with CrCl < 30 mL/min, 44 patients with CrCl 30 to < 50 mL/min, 102 patients with CrCl 50 to < 80 mL/min and 210 patients with CrCl ≥ 80 mL/min

^e^Comorbidities and/or medical history present in ≥ 2% of patients overall are shown individually

^f^Excluding nonmelanoma skin cancer

**Fig. 2:**
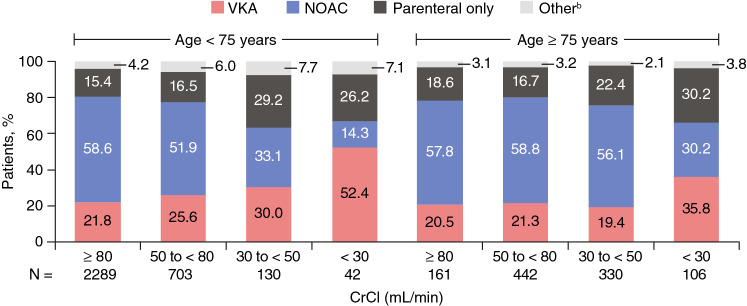
Pattern of anticoagulant use in 4203 patients with age and renal function data available (**a**). *CrCl* creatinine clearance, *NOAC* non-VKA oral anticoagulant, *VKA* vitamin K antagonist. ^a^CrCl data missing for 1892 patients: 1389 patients aged < 75 years and 503 patients aged ≥ 75 years. CrCl estimated using the Cockcroft–Gault formula: < 30 mL/min represents severe impairment, 30 to < 50 mL/min moderate impairment, 50 to < 80 mL/min mild impairment, and ≥ 80 mL/min normal. ^b^“Other” includes catheter-directed or systemic thrombolytic therapy

The prescription patterns of anticoagulants were generally similar between patients aged < 75 and ≥ 75 years who had normal or mild impairment of renal function. Notable differences were apparent between patients aged < 75 and ≥ 75 years who had moderately or severely impaired renal function (Fig. 2). Of those patients with moderate renal impairment aged ≥ 75 years, 56.1% received a NOAC, 19.4% a VKA, and 22.4% parenteral therapy only.

## Discussion

We observed that older patients and those with renal impairment were more often female and more likely to have comorbidities than younger patients or those with normal renal function. Up to 14 days after diagnosis, or by the time of hospital discharge, more than half the patients had been treated with NOACs and over one-fifth were prescribed VKAs. Surprisingly, the proportions of patients receiving NOACs and VKAs were almost the same for the elderly and non-elderly. As expected, NOAC use decreased with worsening renal function, whereas the proportion treated with VKAs tended to increase as renal function declined. It was unexpected, however, that the decrease in the use of NOACs occurred more markedly in younger patients with renal impairment than in elderly patients with renal impairment. Also, the use of parenteral therapy alone increased in patients with moderate and severe renal impairment as compared to patients with normal renal function or mild impairment. This finding is partially unexpected since caution is also recommended with the use of low-molecular-weight heparin in patients with severe renal insufficiency and with the use of fondaparinux in patients with moderate and severe renal insufficiency, due to the renal excretion of these drugs [13]. We note a limitation of the study, that CrCl values were missing for approximately one-third of patients. Data from this and other observational studies show that creatinine levels or estimation of creatinine clearance are not routinely available in patients with VTE. For example, CrCl values were missing for approximately one-fifth of patients in the observational GARFIELD-VTE study cohort [14].

In the real-world setting, patient characteristics and disease management may differ from those in RCTs. The proportions of patients at baseline in RE-COVERY DVT/PE who were aged ≥ 75 years (25.3%) or who had mild-to-moderate renal impairment (38.2%) were greater than those recruited in RCTs of NOACs for acute VTE treatment (~ 12–13% and ~ 26%, respectively) (Table 3) [4, 15, 16]. These RCT data included a pooled analysis of the phase III RE-COVER and RE-COVER II trials of dabigatran versus warfarin analyzed by age and renal function subgroups. In another observational study, GARFIELD-VTE [14], the proportion of patients with CrCl 30–89 mL/min was 41.5% of those with available CrCl estimates. However, fewer patients (18.1%) in the GARFIELD-VTE registry than in RE-COVERY DVT/PE were aged ≥ 75 years (Table 3).

**Table 3:** Comparison of age and renal function in observational trials and randomized controlled trials enrolling patients with acute VTE

	Observational		Randomized		
	RE-COVERY DVT/PE	GARFIELD-VTE [14]	RE-COVER and RE-COVER II pooled [15]	AMPLIFY [4]	Hokusai-VTE [16]
Age					
≥ 75 years, %	25.3	18.1	11.8	14.3	13.4
Mean, years	61.5	60.2^a^	54.8	57.0	55.8
CrCl					
< 30 mL/min, %	3.5^b^	4.6^b^	0.5	0.5	−
30 to < 80 mL/min, %	38.2^b^	41.5^b,c^	26.2	26.0	−
30 to < 50 mL/min, %	10.9^b^	15.7^b,d^	4.6	5.7	6.6
50 to < 80 mL/min, %	27.2^b^	25.8^b,e^	21.5	20.3	−
≥ 80 mL/min, %	58.2^b^	53.9^b,f^	72.4	64.5	−
Missing	Excluded^g^	Excluded^g^	1.0	9.0	−
Mean, mL/min	94.6	−	106.4	−	−

CrCl estimated using the Cockcroft–Gault formula: < 30 mL/min represents severe impairment, 30 to < 50 mL/min moderate impairment, 50 to < 80 mL/min mild impairment and ≥ 80 mL/min normal

*CrCl* creatinine clearance, *DVT*, deep vein thrombosis, *PE* pulmonary embolism, *VTE* venous thromboembolic event

^a^Median

^b^Calculated as percentage of patients with available CrCl data

^c^CrCl 30–89 mL/min

^d^CrCl 30–59 mL/min

^e^CrCl 60–89 mL/min

^f^CrCl ≥ 89 mL/min

^g^Excluded from the calculation of percentages

A key consideration when prescribing any drug in the elderly and in those with impaired renal function, particularly anticoagulants, is reduced drug clearance, as excessive anticoagulation can increase the risk of major bleeding [13, 17]. However, older age [1] and decreased CrCl [18] are also associated with an increased risk of recurrent VTE. Therefore, provision of anticoagulation to these patient groups is challenging. The age and renal function subgroup data from RCTs in VTE show that the effects of NOACs relative to warfarin on safety and efficacy outcomes are consistent with the effects in the entire study population [4–7, 15, 16, 19–21].

Overall, 54.0% of patients in our study were prescribed a NOAC as their oral anticoagulant therapy assessed at 14 days after diagnosis or hospital discharge (whichever was sooner)—greater than the proportion in GARFIELD-VTE (48.7%). Among RE-COVERY DVT/PE patients with moderate renal impairment, about one-third of those aged < 75 years received NOACs. In contrast, more than half of those aged ≥ 75 years were treated with NOACs. The reasons for the different patterns of prescribing among elderly and young patients with renal impairment remain uncertain. However, we speculate that physicians consider that renal impairment is an expected component of frailty among elderly patients, and that the safer profile of NOACs justifies their use despite renal disease. The available subgroup data from RCTs in VTE appear to support this approach [4–7, 15, 16, 19–21], with meta-analyses showing consistent safety and efficacy of NOACs versus VKAs in subgroups including moderate renal impairment and age ≥ 75 years [22, 23].

In summary, the population treated for acute VTE in routine clinical practice includes more elderly and renally impaired patients than those represented in RCTs. Decreasing renal function, particularly in the subgroup with CrCl < 30 mL/min, but not older age, was associated with less use of NOACs. Nevertheless, more than half of the older patients with moderate renal impairment received a NOAC. These baseline data from RE-COVERY DVT/PE provide insight into patient characteristics and how age and renal function are related to patterns of anticoagulant therapy.

## Data Availability

To ensure independent interpretation of clinical study results, Boehringer Ingelheim grants all external authors access to all relevant material, including participant-level clinical study data, and relevant material as needed by them to fulfill their role and obligations as authors under the International Committee of Medical Journal Editors criteria. Furthermore, clinical study documents (e.g., study report, study protocol, statistical analysis plan) and participant clinical study data are available to be shared after publication of the primary manuscript in a peer-reviewed journal and if regulatory activities are complete and other criteria met per the Boehringer Ingelheim Policy on Transparency and Publication of Clinical Study Data: https://trials.boehringer-ingelheim.com/transparency_policy.html. Prior to providing access, documents will be examined and, if necessary, redacted and the data will be de-identified, to protect the personal data of study participants and personnel, and to respect the boundaries of the informed consent of the study participants. Clinical study reports and related clinical documents can be requested via this link: https://trials.boehringer-ingelheim.com/trial_results/clinical_submission_documents.html. All such requests will be governed by a Document Sharing Agreement. Bona fide, qualified scientific and medical researchers may request access to de-identified, analyzable participant clinical study data with corresponding documentation describing the structure and content of the datasets. Upon approval, and governed by a Data Sharing Agreement, data are shared in a secured data-access system for a limited period of 1 year, which may be extended upon request. Researchers should use https://clinicalstudydatarequest.com to request access to study data.
